# Machine Learning-Based Identification of Functional Dysregulation Characteristics in Core Brain Networks of Adolescents with Bipolar Disorder Using Task-fMRI

**DOI:** 10.3390/diagnostics16030466

**Published:** 2026-02-02

**Authors:** Peishan Dai, Ting Hu, Kaineng Huang, Qiongpu Chen, Shenghui Liao, Alessandro Grecucci, Qian Xiao, Xiaoping Yi, Bihong T. Chen

**Affiliations:** 1 School of Computer Science and Engineering, Central South University, Changsha 410083, China; 2 Department of Education, Psychology and Communication Sciences (For.Psi.Com), University of Bari, 70121 Bari, Italy; 3 Clinical Psychology Center of Xiangya Hospital, Central South University, No. 87 Xiangya Road, Changsha 410008, China; 4 Department of Radiology, Chongqing University Three Gorges Hospital, Chongqing University, Chongqing 410008, China; 5 School of Medicine, Chongqing University, Chongqing 400030, China; 6 Clinical Research Center (CRC), Chongqing University Three Gorges Hospital, Chongqing University, Chongqing 404000, China; 7 Medical Pathology Center (MPC), Chongqing University Three Gorges Hospital, Chongqing University, Chongqing 404000, China; 8 Cancer Early Detection and Treatment Center (CEDTC), Chongqing University Three Gorges Hospital, Chongqing University, Chongqing 404000, China; 9 Translational Medicine Research Center (TMRC), Chongqing University Three Gorges Hospital, Chongqing University, Chongqing 404000, China; 10 Department of Diagnostic Radiology, City of Hope National Medical Center, Duarte, CA 91010, USA

**Keywords:** adolescent bipolar disorder, task-based fMRI, machine learning, neuroimaging biomarker, prefrontal cortex, default mode network, mood states

## Abstract

**Background and Objective:** Adolescent bipolar disorder (BD) has substantial symptom overlaps with other psychiatric disorders. Identifying its distinctive candidate neuroimaging markers may be helpful for exploratory early differentiation and to inform future translational studies after independent validation. **Methods:** This cross-sectional study enrolled adolescents with BD and age- and sex-matched healthy controls. Assessments included clinical/behavioral scales and an emotional Go/NoGo task-based fMRI (Go trials require a response; NoGo trials require response inhibition) acquired across three mood states (depression, mania, and remission) and matched controls. We applied several conventional machine learning classifiers to task-fMRI data to classify BD versus healthy controls and to identify the most relevant neuroimaging predictors. **Results:** A total of 43 adolescents with BD (15 in remission, 11 with depression, and 17 with mania) and 43 matched healthy controls were included. Under the Go − NoGo condition, activation-derived features in the remission state showed the strongest discrimination, with RF achieving the best performance (accuracy = 94.29%, AUC = 98.57%). These findings suggest that task-evoked functional alterations may remain detectable during remission. In addition, activation patterns in regions within the limbic system, prefrontal cortex, and default mode network were significantly correlated with clinical scales and behavioral measures implicating these regions in emotion regulation and cognitive functioning in adolescents with BD. **Conclusions:** This study showed that adolescents with BD during remission without manic and depressive symptoms may still have aberrant neural activity in the limbic system, prefrontal cortex, and default mode network, which may serve as a potential candidate neuroimaging signature of adolescent BD.

## 1. Introduction

Bipolar Disorder (BD) is a complex chronic mental disorder that typically manifests in adolescence [1,2,3]. Early diagnosis and intervention are crucial for alleviating symptoms, reducing disease burden, and improving long-term outcomes for patients with BD [3,4]. BD is characterized by distinct mood states, including depressive episodes, manic episodes and remission states, each representing external manifestations of the dynamic evolution of the disease [5,6,7]. Clinically, BD is often misdiagnosed as other mood disorders such as major depressive disorder or emotional disturbance due to similar presentations and overlapping symptoms [8,9,10]. resulting in missing of the optimal treatment window [11]. Moreover, the neuropathological mechanisms of BD are not fully understood, which limits the capability of diagnostic methods for accurate early diagnosis [12].

Brain functional magnetic resonance imaging (fMRI) techniques have been useful for uncovering the neurobiological basis of BD. Unlike resting-state fMRI, which is used to capture spontaneous brain activity [8,9,13,14,15], task-based fMRI assesses brain activation patterns linked to disease states during cognitive or emotional tasks, reducing the interference from spontaneous brain activity [16,17]. Previous studies have identified abnormal functional connectivity in key brain regions such as the default mode network and the limbic system in patients with BD [18,19]. However, most existing task-based fMRI studies have relied on traditional statistical methods (e.g., univariate GLM analysis, voxel-wise group-level *t*-tests/ANOVA, random field theory multiple comparison correction, small-volume ROI analysis, etc.) which are not effective at distinguishing the different disease states (e.g., depressive, manic, and remission phases) in patients with BD, or in classifying individual disease status [20,21,22]. Additionally, although mood-state-specific neuroimaging findings have been reported, cross-state comparability in adolescents remains limited due to small sample sizes and heterogeneous tasks, contrasts, and analytic choices. As a result, studies that systematically evaluate multiple mood states using a unified task-fMRI protocol and a harmonized machine learning framework remain scarce [23]. More studies with advanced analytic approaches that takes into consideration of complex neural activity across different BD states are needed to assess the underlying changes responsible for the drastic shifting of the bipolar states.

Machine Learning (ML) is a data-driven analytical approach capable of detecting patterns and relationships within complex data [24]. which has been used for brain imaging analysis for patients with BD. For instance, machine learning has been used to predict BD episodes, identify BD-related brain regions, and provide neurobiological insights consistent with prior network-level models of BD [25,26,27]. However, the existing machine learning studies are often based on a small sample size, with limited model generalizability [28,29]. In addition, the interpretability of features extracted by these models is inadequate, hindering their direct association with specific neuropathological mechanisms [29,30]. Therefore, it is prudent to expand machine learning applications in BD research and to assist in development of approaches that may inform future translational studies after independent validation.

Here, we used an emotional Go/NoGo task fMRI paradigm to investigate emotion-related, task-evoked brain function abnormalities in adolescents with bipolar disorder (BD) across three mood states (mania, depression, and remission), with age- and sex-matched healthy controls (HCs) as a reference. All participants completed the task fMRI scan and the corresponding neuropsychological scale assessments. We conducted cross-sectional, mood-state-stratified analyses, where the mania, depression, and remission groups were independent cohorts and each BD participant was assigned to only one mood-state subgroup based on the state at the time of scanning. Our main aims were to: (i) classify adolescents with BD in a specific mood state (mania/depression/remission) versus HCs using task-fMRI activation features, and (ii) examine whether discriminative activation patterns differ across mood-state strata. Methodologically, we constructed ROI activation features within a rigorous cross-validation framework and evaluated multiple classification models to ensure reliable performance assessment. To improve clinical interpretability and translational value, we further applied SHAP to attribute model outputs, identify the regional features that contribute most to classification decisions, and test associations between key imaging features and clinical scale scores as well as related clinical variables. Significant associations were reported after multiple-comparison correction, enabling the identification of key brain networks linked to functional abnormalities.

Our hypothesis included the following: there would be differential brain activation patterns on the task-based fMRI between the adolescents with BD and the HC participants, and the task-based functional abnormalities in adolescents with BD may primarily involve key brain regions such as the prefrontal cortex, limbic system, and default mode network, which would be correlated with clinical scales and cognitive measures. This study should contribute to our understanding of neural correlates for adolescent BD across all spectrum of the disease states, which may assist in accurate diagnosis and treatment of adolescent BD.

## 2. Methods

### 2.1. Participants

The data used in this study come from a larger project conducted at Xiangya Hospital of Central South University. All data collection procedures were approved by the Ethics Committee of Xiangya Hospital (approval no. 2022020227). After quality control (Section 2.3), the final analysis included 43 BD participants (15 remission, 11 depression, 17 mania) and 43 age- and sex-matched HCs; 21 of the 64 initially recruited BD participants were excluded after quality control because of excessive motion and/or other data-quality problems, including incomplete fMRI time series and acquisition-related artifacts. All participants were screened to exclude comorbid psychiatric disorders and substance abuse. Medication exposure at the time of scanning was recorded for all patients with BD. This study was approved by the ethics committee, and informed consent was obtained from all participants and their guardians. The demographic information of the data is shown in Table 1.

This was a cross-sectional, state-stratified design; each participant contributed one scan, and no participant was included in more than one mood-state group.

### 2.2. Task-Based fMRI Experimental Design

The task-based Go/NoGo fMRI was performed following the procedural protocol described in Wessa’s study [31], utilizing comparable stimulus sets and trial timing parameters. Stimuli consisted of three facial-expression categories: happy faces (Go), sad faces (NoGo), and neutral faces (Neutral). During the task, participants were instructed to respond on Go (happy) trials and inhibit the response on NoGo (sad) trials, while neutral faces served as a non-emotional comparison condition.

For analysis, we derived six predefined first-level GLM contrasts/conditions from the same task run (Table 2). These contrasts/conditions included four emotional contrasts (Go − NoGo, Go + NoGo, Go − Neutral, and NoGo − Neutral) and two single-condition contrasts (Go and NoGo). To ensure consistent task demands across participants, the trial sequence and timing were kept identical across subjects. The specific contrast definitions are summarized in Table 2.

### 2.3. fMRI Data Acquisition and Preprocessing

MRI acquisition. The task fMRI data were acquired using a T2*-weighted single-shot gradient-echo EPI sequence (TR = 2000 ms, TE = 30 ms, FOV = 240 mm × 240 mm, slice thickness = 4 mm, gap = 0.4 mm, flip angle = 90°, matrix = 64 × 64, 30 slices). For each participant, 310 volumes were collected. The first three volumes were discarded prior to preprocessing to allow for signal stabilization. Before scanning, participants received training on the task. During scanning, stimuli were back-projected from a computer onto a screen and viewed through a mirror mounted above the head coil; stimulus presentation was programmed in E-Prime. Behavioral measures and clinical scales were collected for both adolescents with BD and healthy controls.

Preprocessing and head-motion control. Preprocessing was performed using DPABI/DPARSF (v5.4; SPM-based) [32]. Functional images underwent slice-timing correction (interleaved acquisition; reference slice = 15) and rigid-body realignment. Head motion was quantified using framewise displacement (FD; Jenkinson). Participants with excessive gross motion (maximum translation > 3 mm and/or maximum rotation > 3°) were excluded. For the remaining participants, motion outliers were controlled using volume-wise scrubbing (FD > 0.2 mm), and corresponding scrub regressors were included in nuisance regression. Nuisance regression additionally included white-matter and CSF signals, 24 head-motion regressors (Friston-24 model), and a linear trend. Data were temporally filtered (0.01–0.10 Hz), normalized to MNI space, resampled to 3 × 3 × 3 mm^3^, and spatially smoothed with a Gaussian kernel (FWHM = 4 mm). In total, 21 BD participants were excluded due to data-quality/head-motion considerations; the final sample sizes are reported in Table 1.

### 2.4. Brain Region Activation Under Different Emotional States

A Generalized Linear Model (GLM) was used to characterize task-evoked activation patterns under different emotional conditions. At the first level, we estimated subject-specific contrast z-maps for each comparison condition defined in Table 2. At the second level, for each comparison condition, we fitted a group-level GLM to obtain the corresponding group z-map. Multiple-comparison correction was performed using Gaussian random field (GRF) correction (two-sided; voxel-wise *p* < 0.001 and cluster-wise *p* < 0.05) with a minimum cluster size of 10 contiguous voxels. These GRF-corrected group maps are reported as descriptive activation patterns to summarize condition-specific effects at the cohort level.

Importantly, the whole-sample GRF-corrected maps were used only for reporting/visualization and were not used to construct machine learning features. For machine learning analyses, the second-level GLM and GRF thresholding were repeated within each outer cross-validation fold using TRAIN subjects only, yielding fold-specific abnormal voxel masks to prevent information leakage/circular analysis. These masks were intersected with the AAL atlas to define abnormal AAL ROIs, retaining regions with sufficient overlap (≥10 voxels), which were then used for ROI-level feature extraction (Section 2.5).

### 2.5. ROI Definition and Feature Construction (Abnormal AAL ROIs and ROI Time-Series Features)

Within each outer CV fold, we computed a TRAIN-only second-level group-contrast z-map (the target BD group vs. HCs) and derived a fold-specific abnormal voxel mask by thresholding the z-map using GRF correction (two-sided; voxel-level *p* < 0.001, cluster-level *p* < 0.05; minimum cluster size of 10 contiguous voxels). The abnormal mask was then intersected with the AAL atlas to obtain fold-specific abnormal AAL ROIs, yielding K ROIs for that fold. For each subject, we extracted ROI-mean fMRI time series within these ROIs, producing a subject-level matrix of size $T_{i}\times K$ where $T_{i}$ denotes the available time points. To ensure a fixed input dimensionality without using any information from the test set, we defined $T_{use}={min}_{i}T_{i}^{\left(train\right)}$ within each outer fold based on training subjects only, and truncated all subjects’ ROI time series (TRAIN and TEST) to the first $T_{use}$ time points. The resulting ROI time series were vectorized into a $1\times \left(T_{use}\cdot K\right)$ feature vector per subject. Feature scaling was performed in a leakage-free manner: the scaler was fitted on TRAIN and applied to TEST within each outer fold. Detailed mathematical formulations of ROI-mean extraction and time-series truncation are provided in Supplementary Table S11.

### 2.6. Classification of the Adolescents with BD and the HCs

To evaluate whether activation-derived ROI time-series features can discriminate adolescents with bipolar disorder (BD) from healthy controls (HCs) across the six task contrasts (Table 2), we considered two complementary evaluation settings. First, we performed state-specific classification by comparing each mood-state subgroup (mania, depression, or remission) against matched HCs, using fold-specific ROIs and features derived within each outer CV fold from TRAIN subjects in the corresponding subgroup and TRAIN HCs only. Second, we assessed BD-versus-HC generalization by testing whether ROIs/features derived from a given mood state could generalize to classify pooled BD participants (mania + depression + remission) versus HCs, while keeping ROI definition and all data-dependent processing strictly within TRAIN folds. Classification performance was assessed using nested stratified cross-validation, with an outer 5-fold CV for unbiased evaluation and an inner 3-fold grid-search CV for hyperparameter tuning. All steps that could learn from the data, including ROI definition, $T_{use}$ selection, feature scaling, and model fitting, were confined to TRAIN folds and then applied to the corresponding TEST fold. We evaluated multiple classical classifiers (Logistic Regression, SVM, Random Forest, XGBoost, and Naïve Bayes). The complete hyperparameter search grids for all classifiers are summarized in Supplementary Table S12.

Model explainability was assessed using SHAP: within each outer fold, SHAP values were computed on the held-out test set using an appropriate explainer for the fitted model, and mean absolute SHAP values were aggregated to rank ROI importance. Finally, we pooled out-of-fold predictions from the outer CV and estimated 95% confidence intervals for AUC and accuracy via 10,000 nonparametric bootstrap resamples (excluding resamples containing only one class for AUC computation).

### 2.7. Clinical Correlation and Regression Analysis

In the previous machine learning classification analysis, we identified brain activation patterns under specific contrast conditions that most effectively distinguished adolescents with BD from HC. We hypothesized that these high-discriminability activation patterns are associated with clinical indicators. To test this hypothesis, we performed linear regression analyses linking the identified activation patterns to the collected behavioral measures and clinical scale scores, with the goal of identifying brain regions whose activation profiles are closely related to clinical metrics. To examine clinical relevance, we tested whether the most discriminative activation features were associated with behavioral measures and clinical scale scores. For each contrast and mood-state block, voxelwise regression was performed at the second level and statistical maps were corrected using GRF (two-sided; voxel-wise *p* < 0.001; cluster-wise *p* < 0.05). For clusters surviving GRF correction, we extracted mean regional activation values and fitted linear regression models for each clinical/behavioral metric. We report standardized coefficients (β). Multiple comparisons across region-metric tests were controlled using BH-FDR (q < 0.05) within each contrast/mood-state block.

For clarity, clinical/behavioral association analyses were conducted separately within each group (BD and HC), rather than fitting a single pooled model across all participants. These models were not adjusted for additional covariates (e.g., age, sex, medication status, or head-motion indices) to limit model complexity given the sample size; thus, the findings are interpreted cautiously as exploratory.

## 3. Results

To further evaluate the effectiveness of activation patterns of brain regions under different comparison conditions in distinguishing depressive episodes, manic episodes, and remission states from healthy controls, we used the brain region activation patterns specific to each disease state to identify that particular disease state, as well as BD as a whole.

### 3.1. Brain Activation Patterns for Identifying Corresponding Disease States of BD

The classification performance of brain activation patterns for distinguishing depressive, manic, and remission states of BD from healthy controls (HC) under different comparison conditions is reported in Supplementary Tables S1–S3. Overall, the remission group (Table S2) showed the most stable performance: RF, SVM, and logistic regression achieved AUCs above 90% across most conditions and reached 100% in several cases. In the depression group (Table S1), performance was strong under the Go, Go − NoGo, and Go + NoGo conditions, with RF/SVM/logistic regression frequently achieving an AUC of 100%, whereas XGBoost was generally lower (about 67–83%). In the mania group (Table S3), AUCs were slightly lower than those in the depression and remission groups, but RF/SVM/logistic regression still performed well (about 87–98%). XGBoost showed the largest variability and the lowest AUCs (about 67–87%). In addition, Naïve Bayes achieved high AUCs in all three groups (reaching 100% in some conditions), but its accuracy and sensitivity/specificity varied more across conditions.

### 3.2. Classification Performance Using Remission-Derived Activation Features for Distinguishing BD from Healthy Controls

To evaluate whether activation patterns derived from the remission state can serve as informative features for distinguishing bipolar disorder (BD) from healthy controls (HCs), we trained machine learning models using remission-derived abnormal ROI features obtained from the cross-validated pipeline described in Section 2.5. Cross-validated classification performance across six task-derived contrasts (Go, NoGo, Go − NoGo, Go + NoGo, Go–Neutral, and NoGo–Neutral) using five classical classifiers is summarized in Table 3. In addition, bootstrap 95% confidence intervals for the best-performing remission-derived model are reported in Supplementary Table S6. For BD-versus-HC results using depression-derived and manic-derived features, see Supplementary Tables S4 and S5, respectively.

Among the examined conditions, the Go − NoGo features provided the strongest separation between BD and healthy controls. In this condition, the Random Forest (RF) classifier achieved the best overall performance (Accuracy = 94.29%, Sensitivity = 91.43%, Specificity = 97.14%, AUC = 98.57%). For contrasts involving neutral stimuli, robust discrimination was also observed. Overall, these results suggest that remission-derived activation-related ROI features—particularly those capturing inhibitory-control differences (Go − NoGo)—are informative for distinguishing BD from healthy controls in our cohort. To characterize false-positive/false-negative trade-offs, we also reported PPV (precision), NPV, and FDR derived from confusion matrices (Supplementary Tables S14–S16).

Using pooled out-of-fold predictions from the outer 5-fold cross-validation, we estimated 95% confidence intervals via 10,000 nonparametric bootstrap resamples. For the best-performing setting (Go − NoGo with RF), the pooled out-of-fold AUC was 0.999 (95% CI: 0.995–1.000) and accuracy was 0.971 (95% CI: 0.929–1.000) (Supplementary Tables S4 and S5). Given the modest sample size, these uncertainty estimates should be interpreted cautiously and warrant independent external validation.

**Figure 1:**
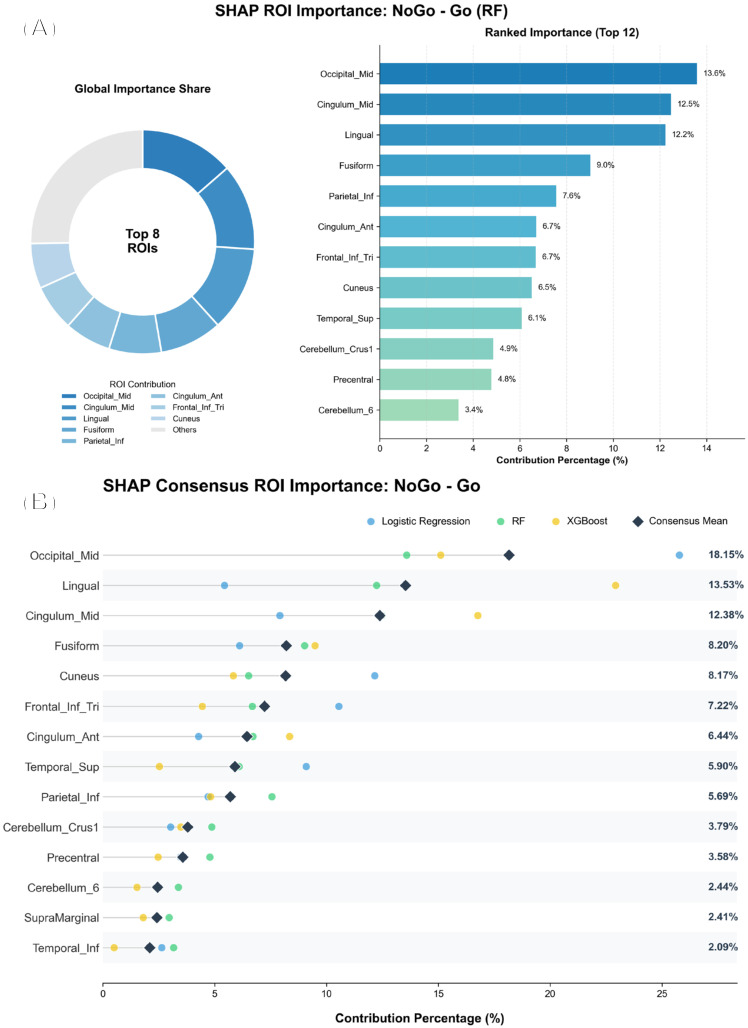
SHAP-based ROI importance for the Go–NoGo classifier (Random Forest). (**A**) The top plot shows the global importance share of the top 8 ROIs. (**B**) The bottom plot shows the ranked contribution percentages for the top 12 ROIs.

As shown in Figure 1, SHAP analysis indicated that the Go − NoGo random forest classifier relied on a compact subset of ROIs rather than distributing importance uniformly across all selected regions. Occipital_Mid contributed the most, followed by Cingulum_Mid and Lingual, with additional contributions from Fusiform and Parietal_Inf. Further informative regions included Cingulum_Ant, Frontal_Inf_Tri, Cuneus, and Temporal_Sup, whereas cerebellar (Cerebellum_Crus1 and Cerebellum_6) and motor-related (Precentral) regions showed comparatively smaller contributions. Collectively, these findings suggest that the discriminative signal captured by the Go − NoGo model is driven primarily by a limited set of abnormal ROIs.

### 3.3. Correlation Analysis Between Activated Brain Regions and Clinical Indicators

The classification data showed that the Go − NoGo task under the remission state achieved 94.29% accuracy, 98.57% sensitivity, 94.05% specificity, and 91.43% area under the curve (AUC) in both the RF models (Table 3). The results show that activated brain regions in the remission group significantly differed from those of the HCs, indicating that the neural activity patterns in adolescent BD may be persistent even during periods of remission.

In the neurocognitive Stroop task, hippocampal and parahippocampal activations were significantly associated with behavioral performance, as reflected by Stroop1 (Supplementary Table S7, Cluster 8: standardized coefficient = −0.363, *p* = 0.005) and Stroop2 (standardized coefficient = −0.316, *p* = 0.030). In addition, the control Go omission rate showed a significant association with activation in the hippocampus/parahippocampal gyrus (Supplementary Table S8, Cluster 11: standardized coefficient = −0.610, *p* = 0.015). Activations in the superior and middle temporal gyri were also significantly correlated with clinical measures, including the Pittsburgh Sleep Quality Index (PSQI; Supplementary Table S7, Cluster 15: standardized coefficient = −0.326, *p* = 0.033) and Trail Making Test Part B (TMT-B; standardized coefficient = −0.611, *p* = 0.022). Furthermore, prefrontal regions (including the superior frontal gyrus, dorsolateral prefrontal cortex, and medial prefrontal cortex) showed significant associations with multiple measures, such as the regeneration task (Supplementary Table S8, Cluster 7: standardized coefficient = −0.724, *p* = 0.021) and the Emotional Go omission rate (standardized coefficient = 0.716, *p* = 0.023).

Overall, linear regression results linking remission-state task-evoked activation patterns (across the Go/NoGo contrasts) to behavioral measures and clinical scales are provided in Supplementary Tables S7 and S8, including cluster sizes and anatomical labels of significant regions. Correlation plots are presented in Supplementary Figures S1–S3. For an overview of multiple-comparison adjustment, BH-FDR-corrected results are summarized in Supplementary Table S9.

## 4. Discussion

In this study, we analyzed task-based fMRI activation patterns and applied ML)models to classify adolescents with BD versus HCs across mood states. We observed abnormal task-evoked activation in the hippocampus, superior temporal gyrus, and prefrontal cortex. Among mood states, remission yielded the strongest BD–HC discrimination, particularly under the Go–NoGo contrast. In addition, activation in key regions was associated with clinical symptom severity and cognitive/behavioral measures.

This study showed that brain region activation patterns from the Go − NoGo task demonstrated optimal performance in machine learning classification of adolescents with BD from the HC participants during the remission state. The remission state in patients with BD, positioned between depressive and manic episodes, represents a relatively stable clinical state, which may better capture trait-like neurobiological features of BD [20]. This study showed that even when emotional symptoms are alleviated, patients may still exhibit neural functional abnormalities as described in the literature [20]. The Go − NoGo task employed in this study was designed to induce responses to various emotional states. Specifically, the “Go” task elicited positive emotional responses, while the “NoGo” task provoked negative emotions, enabling the examination of neural responses to different emotional stimuli [33]. In situations involving emotional conflict, the Go − NoGo task may increase the load on emotional regulation by eliciting emotional responses. Therefore, emotional conflict tasks in remission may provide more discriminative signals between BD and HC in some cohorts, which may partly explain the relatively strong classification performance observed here. The performance gain likely reflects that task-evoked regional activation features capture group-level differences relevant to affective inhibitory control.

Abnormal activation in the hippocampus and parahippocampal gyrus was associated with multiple clinical and behavioral measures (e.g., PSQI, MFQ, Young scores, and neutral Go omission rates). These associations suggest that limbic dysregulation relates to affective symptom severity and task performance in adolescent BD. A prior study also found that abnormal activation in the core components of the limbic system was closely associated with cognitive deficits in patients with BD [32]. These observations further support the involvement of the hippocampus and surrounding regions in emotional and cognitive regulation in BD, emphasizing their critical importance in maintaining stable emotional responses and performing inhibitory tasks.

We observed altered activation in regions commonly linked to the default mode network (DMN), including the superior and middle temporal gyri, during affective regulation. The superior temporal gyrus and middle temporal gyrus are primarily involved in cognitive functions such as self-reflection, episodic memory, and introspection [33,34]. Previous studies have found that patients with BD in remission exhibit significantly reduced activity in the right middle temporal gyrus during resting states, but activity in the bilateral middle temporal gyrus and bilateral superior temporal gyrus is significantly enhanced during emotional and neutral tasks [35]. This suggests that functional abnormalities in the superior temporal gyrus and middle temporal gyrus within the default mode network of the patients with BD may contribute emotional state regulation and the execution of cognitive tasks. In addition, poor sleep quality and reduced cognitive flexibility may further exacerbate difficulties in emotion regulation. The activity levels of these brain regions may contribute to maintaining stable affective and cognitive functioning. We also found that brain regions such as the superior frontal gyrus, dorsolateral prefrontal cortex, and medial prefrontal cortex exhibited significant abnormal activation during emotional tasks in adolescents with BD, which showed significant correlations with multiple clinical scales such as the regeneration task and Emotional Go omission rate. These observations indicated that abnormal activity in the prefrontal cortex was closely related to difficulties in emotional regulation and cognitive control in adolescent patients with BD, consistent with prior studies showing dorsolateral prefrontal cortex being a key brain region for regulating emotional and cognitive function in patients with BD [36,37,38,39].

This study showed extensive neural alterations in key brain networks of the limbic system, prefrontal cortex, and default mode network in adolescent patients with BD. Our findings suggest that abnormal activation within the limbic system may be associated with emotional instability in BD, whereas alterations in prefrontal regions may relate to deficits in cognitive regulation and emotional inhibition. In addition, altered activity in the default mode network may be relevant to self-referential and emotional processing, supporting an association between DMN alterations and cognitive and affective symptoms in BD. Together, these observations suggest that emotional and cognitive dysfunction in BD may be related to alterations across core brain networks, which may inform future hypothesis-driven and longitudinal studies. Even in the absence of acute mood symptoms, patients continued to exhibit abnormal activity in the limbic system, prefrontal cortex, and default mode network, suggesting that such neural alterations may persist across disease stages and may be related to the enduring clinical course of BD. This interpretation is consistent with frameworks proposing that psychiatric disorders may involve persistent systems-level dysregulation even when symptoms subside [40].

## 5. Limitations

Several limitations warrant consideration. First, the modest sample size—particularly after mood-state stratification—requires an exploratory interpretation of the classification performance. We prioritized interpretable classical machine learning over deep learning architectures, as the latter are highly prone to overfitting on high-dimensional clinical datasets of this size. Second, as a single-site study lacking independent external validation, the generalizability of these neural signatures remains to be established. However, while Chen et al. [9] suggested that such medications often ‘normalize’ brain activity, we still observed significant functional dysregulation. This indicates that our findings likely represent core trait markers of adolescent BD rather than treatment effects. Future studies with drug-naïve participants are essential to isolate illness-specific effects from treatment. Finally, the absence of multimodal data (e.g., sMRI/DTI) and longitudinal tracking constrains the comprehensive understanding of neuroprogression. Larger multi-center studies are required to validate these preliminary findings.

## 6. Conclusions

In summary, this study identified mood-state-related alterations in task-evoked brain activity within regions spanning the limbic system, prefrontal cortex, and default mode network, and observed associations with cognitive functioning in adolescents with BD. Using a leakage-controlled cross-validated pipeline, we further evaluated classical machine learning models based on activation-derived ROI time-series features to discriminate BD from healthy controls across emotional task contrasts. These findings suggest that persistent functional dysregulation—particularly during remission—may reflect trait-like neural signatures of adolescent BD. However, the present results should be viewed as exploratory, and independent external validation in larger cohorts will be required before these signatures can be considered clinically predictive or actionable.

## Figures and Tables

**Table 1:** Demographic Information of the study cohort including adolescents with bipolar disorder (BD) and the healthy control (HC) participants.

Participants		Number of Participants	Age (Mean ± SD)	Gender (Male/Female)
Diagnosis	States			
BD	Remission	15	15.33 ± 1.75	7/8
	Depression	11	14.41 ± 1.66	7/4
	Mania	17	14.93 ± 1.77	8/9
HC		43	15.81 ± 1.12	18/25

**Table 2:** First-level contrasts/conditions for the emotional Go/NoGo task.

	Comparison Condition	Interpretation/Definition
Condition 1	Go − NoGo	Happy vs. sad faces (emotion-specific contrast)
Condition 2	Go + NoGo	Combined emotional condition (happy + sad faces; overall emotional-face processing)
Condition 3	Go − Neutral	Happy vs. neutral faces
Condition 4	NoGo − Neutral	Sad vs. neutral faces
Condition 5	Go	Happy (Go) condition only
Condition 6	NoGo	Sad (NoGo) condition only

**Table 3:** Cross-Validated Classification Results Using Remission-Derived ROI Features for Distinguishing BD from Healthy Controls (ROI-mean fMRI time series).

Comparison Condition	Classifier	Accuracy (%)	Sensitivity (%)	Specificity (%)	AUC (%)	F1-Score (%)
Go	RF	84.76	86.07	83.21	92.14	84.67
	SVM	82.10	83.21	81.07	93.27	82.22
	XGBoost	82.00	80.71	83.57	89.49	81.67
	Naive Bayes	83.33	80.71	85.71	89.80	82.69
	Logistic Regression	67.90	66.79	68.93	73.32	67.69
NoGo	RF	88.57	91.43	85.71	95.92	89.22
	SVM	88.57	94.29	82.86	96.33	89.57
	XGBoost	82.86	88.57	77.14	92.65	83.55
	Naive Bayes	90.00	88.57	91.43	95.92	95.92
	Logistic Regression	88.57	91.43	85.71	96.33	89.22
Go − NoGo	RF	**94.29**	**91.43**	**97.14**	**98.57**	**94.05**
	SVM	82.86	85.71	80.00	92.24	82.94
	XGBoost	85.71	85.71	85.71	95.92	85.13
	Naive Bayes	90.00	80.00	100.00	96.73	88.72
	Logistic Regression	85.71	85.71	85.71	94.69	85.66
Go + NoGo	RF	88.57	88.57	88.57	95.51	88.71
	SVM	87.14	91.43	82.86	96.73	87.96
	XGBoost	82.86	80.00	85.71	91.43	82.15
	Naive Bayes	88.57	88.57	88.57	97.78	89.00
	Logistic Regression	88.57	91.43	85.71	97.14	89.39
Go − Neutral	RF	77.58	78.57	76.43	86.47	77.30
	SVM	80.33	78.93	81.79	89.68	79.88
	XGBoost	79.00	81.07	76.79	86.03	78.89
	Naive Bayes	80.33	84.64	76.43	91.92	81.49
	Logistic Regression	80.33	81.79	79.29	89.69	80.36
NoGo − Neutral	RF	88.57	97.14	80.00	98.57	89.67
	SVM	90.00	97.14	82.86	98.37	91.17
	XGBoost	84.29	85.71	82.86	88.57	84.15
	Naive Bayes	90.00	88.57	91.43	95.92	90.03
	Logistic Regression	91.43	97.14	85.71	98.37	92.33

**Note:** Values highlighted in bold indicate the best-performing classifier within each comparison condition (i.e., within each block of five classifiers). **Abbreviations:** BD, bipolar disorder; ROI, region of interest; fMRI, functional magnetic resonance imaging; RF, random forest; SVM, support vector machine; AUC, area under the receiver operating characteristic curve; XGBoost, extreme gradient boosting.

## Data Availability

The data presented in this study are not publicly available due to ethical and privacy restrictions related to human participant data. De-identified data may be made available from the corresponding authors upon reasonable request and with approval from the Ethics Committee of Xiangya Hospital, Central South University.

## Supplementary Materials

The following supporting information can be downloaded at https://www.mdpi.com/article/10.3390/diagnostics16030466/s1, Supplementary Figure S1. Three-view anatomical visualization (A, anterior view) of abnormal brain regions identified in BD patients (BD vs. healthy controls). Supplementary Figure S2. Three-view anatomical visualization (L, left view) of abnormal brain regions identified in BD patients (BD vs. healthy controls). Supplementary Figure S3. Three-view anatomical visualization (P, posterior view) of abnormal brain regions identified in BD patients (BD vs. healthy controls). Supplementary Table S1. Within-depression classification performance across task conditions and classifiers. Supplementary Table S2. Within-remission classification performance across task conditions and classifiers. Supplementary Table S3. Within-manic classification performance across task conditions and classifiers. Supplementary Table S4. BD vs. HC classification performance using depression-derived features across task conditions and classifiers. Supplementary Table S5. BD vs. HC classification performance using manic-derived features across task conditions and classifiers. Supplementary Table S6. Bootstrap 95% confidence intervals for pooled out-of-fold (OOF) performance of the best-performing model (NoGo–Go, Random Forest). Supplementary Table S7. Regression analysis results between significantly activated brain regions in the remission group and BD group. Supplementary Table S8. Brain regions showing significant activation in the remission group, along with remission-group regression outcomes. Supplementary Table S9. Cluster-level associations with clinical and behavioral measures in BD (linear regression): uncorrected p values and BH-FDR–adjusted q values. Supplementary Table S10. Cluster-level associations with clinical and behavioral measures in BD (linear regression): uncorrected p values and BH-FDR–adjusted q values (independent set). Supplementary Table S11. Feature extraction summary (leakage-free, fold-wise). Supplementary Table S12. Hyperparameter grids used in inner-fold grid search (nested CV). Supplementary Table S13. State-of-the-art (SOTA) comparison of machine learning studies for bipolar disorder identification. Supplementary Table S14. Additional clinically interpretable metrics (PPV, NPV, and FDR) for cross-validated classification using remission-state ROI-mean fMRI time-series features (BD vs. healthy controls). Supplementary Table S15. Additional clinically interpretable metrics (PPV, NPV, and FDR) for cross-validated classification using depression-state ROI-mean fMRI time-series features (BD vs. healthy controls). Supplementary Table S16. Additional clinically interpretable metrics (PPV, NPV, and FDR) for cross-validated classification using manic-state ROI-mean fMRI time-series features (BD vs. healthy controls).
