# Supplementary material for: Machine Learning-Based Identification of Functional Dysregulation Characteristics in Core Brain Networks of Adolescents with Bipolar Disorder Using Task-fMRI
Source: Diagnostics (Basel). 2026 Feb 2;16(3):466. doi: 10.3390/diagnostics16030466 (PMC12897290; doi:10.3390/diagnostics16030466)
Supplement: Supplementary file 1 [file diagnostics-16-00466-s001.zip › diagnostics-4136939-supplementary.pdf]

Supplement

S-figure 1-3： illustrates the correlations between the brain region activation patterns of BD patients and clinical scales and behavioral indicators.

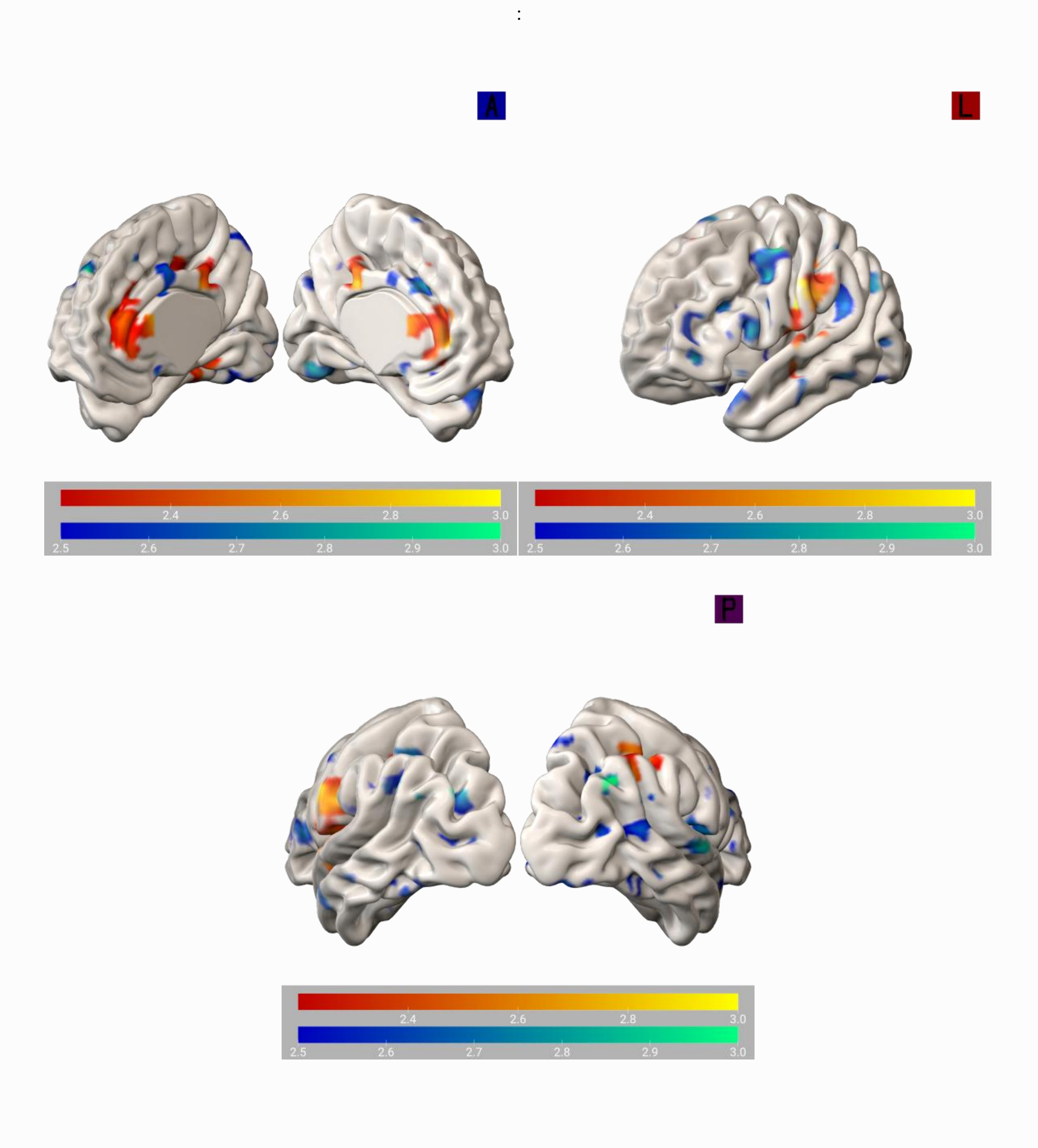

Supplementary Table S1:Within-depression classification performance across task conditions and classifiers

Comparison Condition	Classifier	Accuracy (%)	Sensitivity (%)	Specificity (%)	AUC (%)
Go	RF	95.00	100.00	90.00	100.00
	SVM	91.00	100.00	80.00	100.00
	XGBoost	67.00	60.00	73.33	71.67
	Naive Bayes	83.00	70.00	100.00	100.00
	Logistic Regression	91.00	100.00	80.00	100.00
NoGo	RF	69.00	100.00	40.00	96.67
	SVM	72.00	100.00	43.33	88.33
	XGBoost	55.00	66.67	46.67	68.33
	Naive Bayes	71.00	43.33	100.00	100.00
	Logistic Regression	76.00	100.00	50.00	93.33
NoGo - Go	RF	91.00	100.00	80.00	93.33
	SVM	87.00	100.00	70.00	96.67
	XGBoost	82.00	90.00	73.33	83.33
	Naive Bayes	88.00	80.00	100.00	100.00
	Logistic Regression	87.00	100.00	70.00	96.67
Go + NoGo	RF	86.00	100.00	70.00	100.00
	SVM	91.00	100.00	80.00	93.33
	XGBoost	68.00	66.67	73.33	73.33
	Naive Bayes	82.00	66.67	100.00	100.00
	Logistic Regression	91.00	100.00	80.00	93.33
Go – Neutral	RF	86.00	90.00	80.00	95.00
	SVM	91.00	100.00	80.00	95.00
	XGBoost	55.00	53.33	56.67	71.67
	Naive Bayes	82.00	66.67	100.00	100.00
	Logistic Regression	87.00	90.00	80.00	95.00
NoGo – Neutral	RF	62.00	90.00	33.33	81.67
	SVM	67.00	83.33	50.00	70.00
	XGBoost	55.00	73.33	36.67	66.67
	Naive Bayes	67.00	46.67	90.00	95.00
	Logistic Regression	68.00	93.33	40.00	78.33

Supplementary Table S2: Within-remission classification performance across task conditions and classifiers

Comparison Condition	Classifier	Accuracy (%)	Sensitivity (%)	Specificity (%)	AUC (%)
Go	RF	94.29	95.00	95.00	100.00
	SVM	91.43	95.00	90.00	100.00
	XGBoost	79.52	88.33	71.67	82.78
	Naive Bayes	91.43	85.00	100.00	100.00
	Logistic Regression	94.29	100.00	90.00	100.00
NoGo	RF	91.43	90.00	95.00	100.00
	SVM	88.57	90.00	90.00	100.00
	XGBoost	80.00	78.33	83.33	98.33
	Naive Bayes	91.43	85.00	100.00	100.00
	Logistic Regression	91.43	95.00	90.00	100.00
NoGo - Go	RF	91.43	95.00	90.00	96.67
	SVM	91.43	95.00	90.00	95.00
	XGBoost	79.52	95.00	65.00	88.33
	Naive Bayes	91.43	85.00	100.00	100.00
	Logistic Regression	91.43	95.00	90.00	95.00
Go + NoGo	RF	85.71	83.33	90.00	92.50
	SVM	91.43	95.00	90.00	96.67
	XGBoost	85.24	90.00	83.33	93.33
	Naive Bayes	82.86	78.33	90.00	97.50
	Logistic Regression	94.29	100.00	90.00	96.67
Go – Neutral	RF	79.52	81.67	78.33	90.00
	SVM	91.43	95.00	90.00	98.33
	XGBoost	79.05	86.67	70.00	88.33
	Naive Bayes	88.57	78.33	100.00	96.67
	Logistic Regression	94.29	100.00	90.00	98.33
NoGo – Neutral	RF	74.29	85.00	70.00	97.50
	SVM	82.86	100.00	70.00	93.33
	XGBoost	77.14	73.33	85.00	80.00
	Naive Bayes	91.43	83.33	100.00	100.00
	Logistic Regression	82.86	100.00	70.00	93.33

Supplementary Table S3: Within-manic classification performance across task conditions and classifiers

Comparison Condition	Classifier	Accuracy (%)	Sensitivity (%)	Specificity (%)	AUC (%)
Go	RF	73.33	80.00	66.67	91.11
	SVM	86.67	93.33	80.00	97.77
	XGBoost	73.33	86.67	60.00	86.67
	Naive Bayes	86.67	73.33	100.00	100
	Logistic Regression	90.00	100.00	80.00	97.78
NoGo	RF	76.67	93.33	60.00	91.11
	SVM	70.00	80.00	60.00	86.67
	XGBoost	76.67	73.33	80.00	84.44
	Naive Bayes	83.33	80.00	86.67	97.78
	Logistic Regression	73.33	80.00	66.67	91.11
NoGo - Go	RF	76.67	80.00	73.33	86.67
	SVM	76.67	93.33	60.00	86.67
	XGBoost	73.33	73.33	73.33	82.22
	Naive Bayes	83.33	73.33	93.33	93.33
	Logistic Regression	76.67	93.33	60.00	86.67
Go + NoGo	RF	90.00	100.00	80.00	97.78
	SVM	90.00	100.00	80.00	95.56
	XGBoost	70.00	66.67	73.33	84.44
	Naive Bayes	90.00	80.00	100.00	100.00
	Logistic Regression	90.00	100.00	80.00	93.33
Go – Neutral	RF	86.67	100.00	73.33	95.56
	SVM	90.00	100.00	80.00	95.56
	XGBoost	63.33	66.67	60.00	80.00
	Naive Bayes	90.00	86.67	93.33	96.67
	Logistic Regression	90.00	100.00	80.00	95.56
NoGo – Neutral	RF	80.00	93.33	66.67	90.00
	SVM	83.33	100.00	66.67	97.78
	XGBoost	66.67	80.00	53.33	66.67
	Naive Bayes	90.00	80.00	100.00	100.00
	Logistic Regression	80.00	93.33	66.67	97.78

Supplementary Table S4: BD vs HC classification performance using depression-derived features across task conditions and classifiers

Comparison Condition	Classifier	Accuracy (%)	Sensitivity (%)	Specificity (%)	AUC (%)	F1-Score (%)
Go	RF	80.33	78.93	81.79	89.60	80.24
	SVM	85.75	84.64	87.50	90.40	86.17
	XGBoost	84.25	81.79	86.79	91.34	84.22
	Naive Bayes	83.08	84.64	82.14	91.38	83.69
	Logistic Regression	84.33	84.64	84.29	91.03	84.84
NoGo	RF	72.25	76.43	68.57	84.96	72.55
	SVM	74.92	77.14	73.93	82.77	74.19
	XGBoost	59.33	63.57	55.71	66.70	60.11
	Naive Bayes	81.50	81.43	81.43	88.79	81.44
	Logistic Regression	76.33	79.29	73.93	82.23	76.74
NoGo - Go	RF	82.92	86.43	78.57	85.80	85.60
	SVM	85.58	86.43	83.93	89.24	84.73
	XGBoost	84.17	86.43	80.71	84.78	83.93
	Naive Bayes	78.92	89.29	68.57	86.74	84.34
	Logistic Regression	84.25	84.29	83.57	86.03	88.09
Go + NoGo	RF	86.75	82.14	91.79	94.69	85.60
	SVM	84.25	87.50	81.79	94.46	84.73
	XGBoost	84.17	84.64	84.29	92.05	83.93
	Naive Bayes	84.17	87.14	81.43	91.70	84.34
	Logistic Regression	88.25	87.50	89.64	93.04	88.09
Go - Neutral	RF	77.58	78.57	76.43	86.47	77.30
	SVM	80.33	78.93	81.79	89.69	79.88
	XGBoost	79.00	81.07	76.79	86.03	78.89
	Naive Bayes	80.33	84.64	76.43	91.92	81.49
	Logistic Regression	80.33	81.79	79.29	89.69	80.36
NoGo - Neutral	RF	73.67	78.93	68.57	80.69	74.45
	SVM	78.92	89.64	68.21	84.64	81.01
	XGBoost	71.00	76.07	66.07	75.27	72.10
	Naive Bayes	76.33	89.64	63.21	84.02	79.21
	Logistic Regression	80.17	86.79	73.57	84.64	81.19

Supplementary Table S5: BD vs HC classification performance using manic-derived features across task conditions and classifiers

Comparison Condition	Classifier	Accuracy (%)	Sensitivity (%)	Specificity (%)	AUC (%)	F1-Score (%)
Go	RF	79.14	91.43	66.85	89.85	81.79
	SVM	91.71	88.57	94.85	95.82	91.13
	XGBoost	81.81	91.43	72.19	89.69	83.29
	Naive Bayes	74.95	85.71	64.19	85.66	77.64
	Logistic Regression	72.10	74.64	69.59	80.00	71.96
NoGo	RF	80.48	94.64	66.07	91.96	83.44
	SVM	73.52	91.79	55.36	85.20	77.86
	XGBoost	70.67	80.36	61.43	79.64	73.79
	Naive Bayes	90.38	83.93	97.50	97.09	88.60
	Logistic Regression	79.14	91.79	66.43	86.22	82.39
NoGo - Go	RF	79.24	80.00	78.21	82.19	78.80
	SVM	79.05	85.71	72.86	85.92	80.19
	XGBoost	73.71	74.64	72.50	80.61	72.94
	Naive Bayes	83.24	85.71	80.71	88.67	82.32
	Logistic Regression	83.24	82.86	83.93	84.44	82.38
Go + NoGo	RF	87.71	87.14	89.29	99.59	86.64
	SVM	93.24	95.00	92.14	98.52	93.31
	XGBoost	89.05	89.64	89.29	99.59	88.69
	Naive Bayes	90.48	89.64	92.14	98.52	90.03
	Logistic Regression	89.05	92.14	86.79	97.81	89.42
Go - Neutral	RF	84.76	86.07	83.45	92.14	84.67
	SVM	82.10	83.21	80.99	93.27	82.22
	XGBoost	82.00	80.71	83.29	89.49	81.67
	Naive Bayes	83.33	80.71	85.95	89.80	82.69
	Logistic Regression	67.90	66.79	69.01	73.32	67.69
NoGo - Neutral	RF	79.05	83.21	74.64	91.38	79.88
	SVM	77.81	76.07	80.36	90.00	76.19
	XGBoost	84.67	86.07	83.21	86.94	85.25
	Naive Bayes	81.81	88.57	74.64	89.59	82.71
	Logistic Regression	80.38	85.71	74.64	89.59	81.32

Supplementary Table S6:Bootstrap 95% confidence intervals for pooled out-of-fold (OOF) performance of the best-performing model (NoGo–Go, Random Forest)

contrast	classifier	metric	mean	ci95_low	ci95_high	n_valid	n_boot
NoGo-Go	RF	AUC	0.9991	0.995	1	10000	10000
		Accuracy	0.9711	0.9287	1	10000	10000

Note: Two AUC aggregation schemes are reported. In Table 3, AUC is the mean of fold-wise test AUCs from the outer cross-validation (computed within each outer test fold and then averaged). In Supplementary Table S6, AUC is the pooled out-of-fold

(OOF) AUC computed by concatenating all outer-fold OOF predicted probabilities, with 95% CIs estimated via 10,000 bootstrap resamples. Given the modest sample size and potential fold-to-fold variability in score distributions, these two aggregation schemes may yield different AUC values. For consistency in model comparison, we primarily refer to the outer-fold mean AUC in Table 3, and use Table S6 to quantify uncertainty of the pooled OOF performance.

Supplementary Table S7: Regression Analysis Results Between Significant Activated Brain Regions in Remission Group and BD Group

**BD / Linear Regression Brain Regions: voxel-level**			coefficient			coefficient			coefficient			coefficient	
Middle Occipital Gyrus: 26<br>Middle Temporal Gyrus: 30	Cluster 1	MFQ	Normalization factor	0.346	Digit Span Backward	Normalization factor	0.309	Emotional Go Omission Rate	Normalization factor	0.312			
			p-value	0.025		p-value	0.049		p-value	0.038			
Medial and Paracingulate Gyri: 405<br>Paracentral Lobule: 10	Cluster 2	Digit Span Backward	Normalization factor	0.246									
			p-value	0.020									
Frontal_Sup _L: 82，	Cluster 3	PSQI	Normalization factor	-0.313	regeneration	Normalization factor	0.412						
			p-value	0.040		p-value	0.006						
Superior Occipital Gyrus_R: 38，<br>Middle Occipital Gyrus_R: 4，<br>Cuneus_R: 4，	Cluster 4	PSQI	Normalization factor	-0.360	regeneration	Normalization factor	0.346	Digit Span Backward	Normalization factor	0.322		w	
			p-value	0.0170		p-value	0.022		p-value	0.039			
Frontal_Sup _L: 62，	Cluster 5	Digit Span Backward	Normalization factor	0.422	Neutral Emotional Go Omission Rate	Normalization factor	-0.347				w		
			p-value	0.011		p-value	0.032						
Cerebellum_Crus2_R: 78，<br>Cerebellum_Crus1_R: 12，<br>Cerebellum_8_R: 10，<br>Cerebellum_7b_R: 5，	Cluster 6	stroop 3	Normalization factor	-0.300	Neutral NOGO error rate	Normalization factor	-0.417	Emotional nogo minus neutral	Normalization factor	0.340	Emotional GO reaction time	Normalization factor	-0.327
			p-value	0.037		p-value	0.010		p-value	0.036		p-value	0.048

								nogo					
Superior Parietal Lobule_L: 10，Postcentral Gyrus_L: 39，	Cluster 7	MFQ	Normalization factor	-0.333	Emotional nogo error rate	Normalization factor	-0.340	Neutral NOGO error rate	Normalization factor	-0.340	NoGo error rate	Normalization factor	-0.310
			p-value	0.033		p-value	0.033		p-value	0.033		p-value	0.045
Parahippocampal gyrus _R: 42，The hippocampus _R: 2，	Cluster 8	Stroop 1	Normalization factor	-0.363	stroop2	Normalization factor	-0.316						
			p-value	0.005		p-value	0.030						
Superior temporal gyrus _R: 187，Supramarginal Gyrus _R: 184，Postcentral gyrus _R: 10，Rolandic operculum _R: 30，	Cluster 9	YOUNG	Normalization factor	-0.347	Digit Span Backward	Normalization factor	0.372						
			p-value	0.025		p-value	0.016						
Precentral gyrus _R: 43，	Cluster 10	PSQI	Normalization factor	-0.444	Stroop1	Normalization factor	-0.292	Stroop2	Normalization factor	-0.428	Neutral NOGO error rate	Normalization factor	-0.311
			p-value	0.003		p-value	0.024		p-value	0.002		p-value	0.048
Supramarginal Gyrus: 114，Precentral gyrus _L: 226，Frontal_Sup _L: 64，Inferior frontal gyrus, triangular part: 158，	Cluster 11	stroop3	Normalization factor	0.42	Trail Making Test (TMT) Part A	Normalization factor	-0.311	Digit Span Forward	Normalization factor	0.308	Digit Span Backward	Normalization factor	0.368
			p-value	0.036		p-value	0.045		p-value	0.032		p-value	0.018
Dorsolateral superior frontal gyrus _R: 1 Supplemental exercise area _R: 152 Medial and paracingulate gyrus _R:296 Anterior and paracingulate gyrus _R:153 Medial superior frontal gyrus _R: 13，Supplemental exercise area _L: 126，	Cluster 12	Digit Span Backward	Normalization factor	0.321									
			p-value	0.040									

Medial and paracingulate gyrus_L: 405<br>Medial superior frontal gyrus_L: 41<br>Anterior cingulate and paracingulate gyrus_L: 186													
Inferior triangular frontal gyrus_R: 276，<br>Frontal_R: 42，	Cluster 13	stroop 3	Normalization factor	0.291									
			p-value	0.034									
Superior temporal gyrus_L: 194，<br>supramarginal gyrus_L: 47，	Cluster 14	PSQI	Normalization factor	-0.315	MFQ	Normalization factor	0.372	YOUNG	Normalization factor	-0.483	TMTB	Normalization factor	-0.336
			p-value	0.048		p-value	0.020		p-value	0.002		p-value	0.037
		Emotional GO reaction time	Normalization factor	0.496	Neutral GO reaction time	Normalization factor	0.538	Control GO reaction time	Normalization factor	0.438			
			p-value	0.002		p-value	0.001		p-value	0.006			
Middle Temporal Gyrus_R: 69，<br>Superior temporal gyrus _R: 322，<br>Transverse Temporal Gyrus_R: 3，	Cluster 15	PSQI	Normalization factor	-0.326	TMTB	Normalization factor	-0.611						
			p-value	0.033		p-value	0.022						
Dorsolateral superior frontal gyrus _R: 89，<br>Medial superior frontal gyrus _R: 34，	Cluster 16	regeneration	Normalization factor	-0.491									
			p-value	0.001									
Frontal_R: 63，<br>Dorsolateral superior frontal gyrus _R: 2，	Cluster 17	TMTB	Normalization factor	0.687	Emotional GO reaction time	Normalization factor	-0.357						
			p-value	0.005		p-value	0.031						
Cerebellum_4_5_L: 38，<br>Cerebellum_6_L: 259，<br>Cerebellum_Crus1_L: 123，	Cluster 18	PSQI	Normalization factor	-0.332									

Fusiform Gyrus_L: 13， Inferior Temporal Gyrus_L: 11，	p-value	0.029

Supplementary Table S8 Brain regions showing significant activation in the remission group, along with remission group regression outcomes

BD / Linear Regression Brain Regions: voxel-level			coefficient			coefficient			coefficient			coefficient	
lingual gyrus _L: 46， Parahippocampal gyrus _L: 37， Hippocampus _L: 12，	Cluster 1	regeneration	Normalization factor	0.706									
			p-value	0.004									
Superior temporal gyrus _R: 187， Supramarginal Gyrus _R: 184， Postcentral gyrus _R: 10， Rolandic operculum _R: 30，	Cluster 2	Stroop3	Normalization factor	0.046	Digit Span Backward	Normalization factor	0.372						
			p-value	0.042		p-value	0.021						
Supramarginal Gyrus: 114， Precentral gyrus _L: 226， Frontal_Sup _L: 64， Inferior frontal gyrus, triangular part: 158，	Cluster 3	PSQI	Normalization factor	-0.593	Trail Making Test (TMT) Part A	Normalization factor	-0.627	Digit Span Forward	Normalization factor	0.541	Digit Span Backward	Normalization factor	0.541
			p-value	0.019		p-value	0.011		p-value	0.023		p-value	0.023
The hippocampus _R: 49， Amygdala _R: 7， Parahippocampal gyrus _R: 1，	Cluster 4	Digit Span Backward	Normalization factor	-0.048									
			p-value	0.017									
Precentral gyrus _R: 43，	Cluster 5	PSQI	Normalization factor	-0.494	Neutral Emotional Go Omission Rate	Normalization factor	-0.551						
			p-value	0.046		p-value	0.033						
Orbital Part of the Inferior Frontal Gyrus_L: 68， Superior temporal gyrus_L: 168， Insular Cortex_L: 279， Putamen_L: 1，	Cluster 6	stroop3	Normalization factor	0.207	regeneration	Normalization factor	0.557						

Inferior frontal gyrus, triangular part: 48，<br>Supramarginal Gyrus: 54，<br>Operculum_L: 34，<br>Superior temporal gyrus_L: 37，<br>Middle Temporal Gyrus_L: 5，<br>Undefined: 4，			p-value	0.008		p-value	0.034
Dorsolateral superior frontal gyrus _R: 66，<br>Medial superior frontal gyrus _R: 79，<br>Anterior and paracingulate gyrus _R: 7，<br>Medial superior frontal gyrus_L: 290，<br>Anterior cingulate and paracingulate gyrus_L: 196，<br>Orbital Part_L: 54，<br>Dorsolateral Superior Frontal Gyrus _L: 98，	Cluster 7	regeneration	Normalization factor	-0.724	Emotional Emotional Go Omission Rate	Normalization factor	0.716
			p-value	0.021		p-value	0.023
Superior Parietal Lobule_R: 5，<br>Precuneus_R: 314，<br>Pericalcarine Cortex_R: 17，<br>Cuneus_R: 3，<br>Medial and paracingulate gyrus _R: 42，<br>Posterior Cingulate Cortex_R: 42，<br>Vermis_4_5: 51，<br>Precuneus_L: 307，<br>Pericalcarine Cortex_L: 57，<br>Cuneus_L: 1，<br>Posterior Cingulate Cortex_L: 232，<br>Medial and paracingulate gyrus_L: 88，<br>Cerebellum_4_5_L: 5，<br>lingual gyrus _L: 19，	Cluster 8	TMT B	Normalization factor	-0.564			
			p-value	0.022			
Cerebellum_4_5_L: 38，<br>Cerebellum_6_L: 259，<br>Cerebellum_Crus1_L: 123，<br>Fusiform Gyrus_L: 13，<br>Inferior Temporal Gyrus_L: 11，	Cluster 9	PSQI	Normalization factor	-0.501			
			p-value	0.048			

Frontal_Sup _L: 62，	Cluster 10	Digit Span Backward	Normalization factor	0.323	Neutral Emotional Go Omission Rate	Normalization factor	-0.515
			p-value	0.020		p-value	0.041
Parahippocampal gyrus _R: 42，<br>The hippocampus _R: 2，	Cluster 11	Emotional Go Omission Rate	Normalization factor	-0.610			
			p-value	0.015			
Superior temporal gyrus_L: 194，<br>supramarginal gyrus_L: 47，	Cluster 12	stroop3	Normalization factor	-0.173	TMT B	Normalization factor	-0.611
			p-value	0.044		p-value	0.022

Supplementary Table S9:Cluster-level associations with clinical and behavioral measures in BD (linear regression): uncorrected p values and BH-FDR–adjusted q values

cluster	predictor	coefficient	p_value	q_bh_fdr
1	MFQ	0.346	0.025	0.047
1	Emotional Go Omission Rate	0.312	0.038	0.047
1	Digit Span Backward	0.309	0.049	0.049
3	regeneration	0.412	0.006	0.024
3	PSQI	-0.313	0.04	0.047
5	Digit Span Backward	0.422	0.011	0.036
5	Neutral Emotional Go Omission Rate	-0.347	0.032	0.047
6	Neutral NOGO error rate	-0.417	0.01	0.036
6	Emotional nogo minus neutral nogo	0.34	0.036	0.047
6	stroop3	-0.3	0.037	0.047
6	Emotional GO reaction time	-0.327	0.048	0.049
7	Emotional nogo error rate	-0.34	0.033	0.047
7	MFQ	-0.333	0.033	0.047
7	Neutral NOGO error rate	-0.34	0.033	0.047
7	NoGo error rate	-0.31	0.045	0.049
8	Stroop1	-0.363	0.005	0.024
8	stroop2	-0.316	0.03	0.047
10	Stroop2	-0.428	0.002	0.016
10	PSQI	-0.444	0.003	0.020
10	Stroop1	-0.292	0.024	0.040
10	Neutral NOGO error rate	-0.311	0.048	0.049
11	Digit Span Backward	0.368	0.018	0.047
11	Digit Span Forward	0.308	0.032	0.047
11	stroop3	0.42	0.036	0.047
11	Trail Making Test (TMT) Part A	-0.311	0.045	0.049
12	Digit Span Backward	0.321	0.04	0.047
13	stroop3	0.291	0.034	0.047
14	Neutral GO reaction time	0.538	0.001	0.016
14	Emotional GO reaction time	0.496	0.002	0.016
14	YOUNG	-0.483	0.002	0.016
14	Control GO reaction time	0.438	0.006	0.024
14	MFQ	0.372	0.02	0.047
14	TMT B	-0.336	0.037	0.047
14	PSQI	-0.315	0.048	0.049
15	TMT B	-0.611	0.022	0.047
15	PSQI	-0.326	0.033	0.047
16	regeneration	-0.491	0.001	0.016
17	TMT B	0.687	0.005	0.024
17	Emotional GO reaction time	-0.357	0.031	0.047
18	PSQI	-0.332	0.029	0.047

Supplementary Table S10:Cluster-level associations with clinical and behavioral measures in BD (linear regression): uncorrected p values and BH-FDR–adjusted q values (independent set)

cluster	predictor	coefficient	p_value	q_bh_fdr
1	regeneration	0.706	0.004	0.033
2	Digit Span Backward	0.372	0.021	0.033
2	Stroop3	0.046	0.042	0.046
3	Trail Making Test (TMT) Part A	-0.627	0.011	0.033
3	PSQI	-0.593	0.019	0.033
3	Digit Span Backward	0.541	0.023	0.033
3	Digit Span Forward	0.541	0.023	0.033
5	Neutral Emotional Go Omission Rate	-0.551	0.033	0.043
5	PSQI	-0.494	0.046	0.046
6	stroop3	0.207	0.008	0.033
6	regeneration	0.557	0.034	0.043
7	regeneration	-0.724	0.021	0.033
7	Emotional Emotional Go Omission Rate	0.716	0.023	0.033
8	TMT B	-0.564	0.022	0.033
10	Digit Span Backward	0.323	0.02	0.033
10	Neutral Emotional Go Omission Rate	-0.515	0.041	0.046
11	Emotional Go Omission Rate	-0.61	0.015	0.033
12	TMT B	-0.611	0.022	0.033
12	stroop3	-0.173	0.044	0.046

Supplementary Table S11. Feature Extraction Summary (Leakage-Free, Fold-wise)

Extracted features / Step	Mathematical formulation	Explanation (replicable details)
Fold-specific abnormal ROI set (AAL-level)	$R^{(f)} = \{r_k\}_{\{k=1..k_f\}}$, $r_k = AAL_k \cap M^{(f)}$	In outer fold f, an abnormal voxel mask $M^{(f)}$ is derived using TRAINING data only (GRF-thresholded second-level group-contrast map) and intersected with the AAL atlas to yield a fold-specific set of abnormal ROIs. k_f may vary across folds.
Time-series preprocessing (within-subject)	$y'_{i(v,t)} = zscore(detrend(y_{i(v,t)}))$	Within each subject, voxelwise BOLD time series were detrended and z-standardized prior to ROI averaging (implemented via a masker with detrend=True and standardize=True; optional band-pass can be enabled if specified).
ROI-mean fMRI time series	$x_i^{(f)}(t,k) = \frac{1}{\lvert r_k^{(f)} \rvert} \sum_{v \in r_k^{(f)}} y'_i(v,t)$ $x_i^{(f)} \in \mathbb{R}^{T_1 \times K_f}$	For subject i and ROI r_k in fold f, compute the voxel-averaged BOLD signal over ROI voxels, producing a subject-level matrix $x_i^{(f)} \in \mathbb{R}^{T_1 \times K_f}$.
Fixed-length truncation (training-defined)	$T_{use}^{(f)} = \min_{i \in Train(f)} (T_i)$	To ensure a fixed input dimensionality without test-set information, $T_{use}^{(f)}$ is defined using training subjects only. Both training and test subjects are truncated accordingly.
Time-series truncation	$\tilde{X}_i^{(f)} = X_i^{(f)}[0{:}\mathrm{T}_{\mathrm{use}}^{(\mathrm{f})}, :]$	Truncate each subject’s ROI time-series matrix to the first $\mathrm{T}_{\mathrm{use}^{(\mathrm{f})}}$ time points.
Vectorized feature vector	$v_i^{(f)} = vec(\tilde{X}_i^{(f)}) \in \mathbb{R}^{\{T_{use}^{(f)} \cdot k_f\}}$	Flatten the truncated ROI time-series matrix into a one-dimensional feature vector (a fixed and consistent ordering is used across folds).
Leakage-free feature scaling	$z_i^{(f)} = (v_i^{(f)} - \mu^{(f)} \sigma^{(f)})$	Feature standardization is performed using mean $\mu^{(f)}$ and standard deviation $\sigma^{(f)}$ estimated from the TRAINING split only, and then applied to the corresponding test split.
Final machine-learning input	$z_i^{(f)}$	The standardized vector $z_i^{(f)}$ is used as the final input to the classifier in outer fold f.

Abbreviations: f denotes an outer cross-validation fold; TRAIN(f) and TEST(f) denote the training and test splits in fold f; AAL denotes the Automated Anatomical Labeling atlas.

Supplementary Table S12. Hyperparameter grids used in inner-fold grid search (nested CV)

**Model**	**Hyperparameters (grid)**
Model	Hyperparameters (grid)
Logistic Regression (LR)	C ∈ {0.01, 0.1, 1, 10, 100}; penalty = l2; solver = liblinear
Support Vector Machine (SVM)	kernel = linear (fixed); C ∈ {0.01, 0.1, 1, 10, 100}
Random Forest (RF)	n_estimators ∈ {400, 800}; max_depth ∈ {None, 10, 20}
XGBoost (XGB)	n_estimators ∈ {300, 500}; learning_rate ∈ {0.05, 0.1}; max_depth ∈ {3, 5}; subsample = 0.8; colsample_bytree = 0.8

Note: Hyperparameter tuning was performed using an inner 3-fold cross-validation within each outer-fold training split (nested

CV). The configuration maximizing the mean inner-fold AUC was selected and then applied to the corresponding held-out outer-fold test set.

Supplementary Table 13: State-of-the-Art (SOTA) Comparison of Machine Learning Studies for Bipolar Disorder Identification

Study (Reference)	Imaging/Modality	Classification Target	Method	Sample Size	Best Accuracy	Scientific Significance & Research Gap
Wang et al. [15]	rs-fMRI (Resting-state)	BD-II vs. Healthy Controls	Radiomics + SVM	207 (90 BD, 117 HC)	80.5%	Distinguished unmedicated adult BD-II using resting-state radiomics; however, lacked focus on adolescents and task-evoked dynamic functions.
Mateo-Sotos et al.[25]	EEG (Brain Electricity)	BD vs. Depression Patients	XGBoost	Clinic Records	94.0%	Achieved high accuracy using EEG signals; however, low spatial resolution limits the precise localization of brain network dysfunctions.
Jiang et al. [27]	rs-fMRI (Functional Conn.)	Misdiagnosed BD vs. Unipolar Depression	Amygdala FC + SVM	117 (48 BD, 69 UD)	81.0%	Addressed misdiagnosis through amygdala connectivity in pre-manic patients; however, did not evaluate feature stability during remission.
This Study (2026)	Task-fMRI (Go/NoGo)	Adolescent BD vs. Healthy Controls	Random Forest + SHAP	86 (43 BD, 43 HC)	94.29%	Integrates task-evoked features with explainable AI (SHAP), achieving top-tier performance for adolescent BD with superior spatial and clinical interpretability.

Table S14: Additional Clinically Interpretable Metrics (PPV, NPV, and FDR) for Cross-Validated Classification Using Remission-State ROI-Mean fMRI Time-Series Features (BD vs. Healthy Controls)

Comparison Condition	Classifier	PPV(Precision)%	NPV%	FDR%
Go	RF	83.68	85.66	16.32
	SVM	81.47	82.84	18.53
	XGBoost	83.09	81.25	16.91
	Naive Bayes	84.96	81.63	15.04
	Logistic Regression	68.25	67.49	31.75
NoGo	RF	86.50	91.11	13.50
	SVM	84.98	93.62	15.02
	XGBoost	79.50	87.08	20.50
	Naive Bayes	91.07	89.83	8.93
	Logistic Regression	86.50	91.11	13.50
NoGo - Go	RF	96.97	91.96	3.03
	SVM	81.82	84.85	18.18
	XGBoost	85.71	85.71	14.29
	Naive Bayes	100.00	83.93	0.00
	Logistic Regression	85.71	85.71	14.29
Go + NoGo	RF	88.57	88.57	11.43
	SVM	84.21	90.62	15.79
	XGBoost	84.86	81.63	15.14
	Naive Bayes	88.57	88.57	11.43
	Logistic Regression	86.49	90.91	13.51
Go - Neutral	RF	76.85	78.14	23.15
	SVM	81.25	79.39	18.75
	XGBoost	77.75	80.43	22.25
	Naive Bayes	78.49	83.29	21.51
	Logistic Regression	79.68	81.86	20.32
NoGo - Neutral	RF	82.93	96.55	17.07
	SVM	84.99	96.77	15.01
	XGBoost	83.70	85.29	16.30
	Naive Bayes	91.07	88.24	8.93
	Logistic Regression	87.18	96.77	12.82

Table S15: Additional Clinically Interpretable Metrics (PPV, NPV, and FDR) for Cross-Validated Classification Using Depression-State ROI-Mean fMRI Time-Series Features (BD vs. Healthy Controls)

Comparison Condition	Classifier	PPV(Precision)%	NPV%	FDR%
Go	RF	81.25	79.52	18.75
	SVM	87.13	85.07	12.87
	XGBoost	86.09	82.66	13.91
	Naive Bayes	82.58	84.25	17.42
	Logistic Regression	84.34	84.59	15.66
NoGo	RF	70.85	74.28	29.15
	SVM	74.75	76.71	25.25
	XGBoost	58.95	62.14	41.05
	Naive Bayes	81.43	81.43	18.57
	Logistic Regression	75.29	78.32	24.71
NoGo - Go	RF	80.12	85.32	19.88
	SVM	84.32	85.80	15.68
	XGBoost	81.75	85.68	18.25
	Naive Bayes	74.00	88.46	26.00
	Logistic Regression	83.42	84.57	16.58
Go + NoGo	RF	90.93	83.77	9.07
	SVM	83.10	86.67	16.90
	XGBoost	84.45	84.50	15.55
	Naive Bayes	83.02	86.41	16.98
	Logistic Regression	89.39	87.93	10.61
Go - Neutral	RF	76.85	78.14	23.15
	SVM	81.25	79.39	18.75
	XGBoost	77.75	80.43	22.25
	Naive Bayes	78.49	83.29	21.51
	Logistic Regression	79.68	81.86	20.32
NoGo - Neutral	RF	71.51	77.75	28.49
	SVM	73.82	86.50	26.18
	XGBoost	69.16	74.02	30.84
	Naive Bayes	70.92	87.64	29.08
	Logistic Regression	76.66	84.92	23.34

Table S16: Additional Clinically Interpretable Metrics (PPV, NPV, and FDR) for Cross-Validated Classification Manic-State ROI-Mean fMRI Time-Series Features (BD vs. Healthy Controls)

Comparison Condition	Classifier	PPV(Precision)%	NPV%	FDR%
Go	RF	73.39	88.64	26.61
	SVM	94.50	89.25	5.50
	XGBoost	76.68	89.39	23.32
	Naive Bayes	70.53	81.79	29.47
	Logistic Regression	71.05	73.29	28.95
NoGo	RF	73.59	92.81	26.41
	SVM	67.26	87.10	32.74
	XGBoost	67.56	75.75	32.44
	Naive Bayes	97.49	85.81	2.51
	Logistic Regression	73.24	91.59	26.76
NoGo - Go	RF	78.59	79.64	21.41
	SVM	75.97	84.01	24.03
	XGBoost	73.09	74.08	26.91
	Naive Bayes	81.68	84.54	18.32
	Logistic Regression	83.83	82.99	16.17
Go + NoGo	RF	89.08	87.33	10.92
	SVM	92.45	94.78	7.55
	XGBoost	89.26	89.67	10.74
	Naive Bayes	92.01	89.78	7.99
	Logistic Regression	87.57	91.41	12.43
Go - Neutral	RF	83.98	85.67	16.02
	SVM	81.65	82.76	18.35
	XGBoost	82.89	81.09	17.11
	Naive Bayes	85.21	81.43	14.79
	Logistic Regression	71.46	64.33	28.54
NoGo - Neutral	RF	76.62	81.65	23.38
	SVM	79.46	77.14	20.54
	XGBoost	83.73	85.59	16.27
	Naive Bayes	78.97	87.02	21.03
	Logistic Regression	78.19	84.17	21.81
